# Antisense-acting riboswitches: A poorly characterized yet important model of transcriptional regulation in prokaryotic organisms

**DOI:** 10.1371/journal.pone.0281744

**Published:** 2023-02-21

**Authors:** Mariela Serrano-Gutiérrez, Enrique Merino

**Affiliations:** Departamento de Microbiología Molecular, Instituto de Biotecnología, Universidad Nacional Autónoma de México, Cuernavaca, Morelos, México; University of Montana, UNITED STATES

## Abstract

Riboswitches are RNA elements involved in regulating genes that participate in the biosynthesis or transport of essential metabolites. They are characterized by their ability to recognize their target molecules with high affinity and specificity. Riboswitches are commonly cotranscribed with their target genes and are located at the 5’ end of their transcriptional units. To date, only two exceptional cases of riboswitches being situated at the 3’ end and transcribing in the antisense direction of their regulated genes have been described. The first case involves a SAM riboswitch located at the 3’ end of the *ubiG-mccB-mccA* operon in *Clostridium acetobutylicum* involved in converting methionine to cysteine. The second case concerns a Cobalamin riboswitch in *Listeria monocytogenes* that regulates the transcription factor PocR related to this organism’s pathogenic process. In almost a decade since the first descriptions of antisense-acting riboswitches, no new examples have been described. In this work, we performed a computational analysis to identify new examples of antisense-acting riboswitches. We found 292 cases in which, according to the available information, we infer that the expected regulation of the riboswitch is consistent with the signaling molecule it senses and the metabolic function of the regulated gene. The metabolic implications of this novel type of regulation are thoroughly discussed.

## Introduction

Riboswitches are RNA elements involved in the regulation of certain types of genes that participate in the biosynthesis or transport of essential metabolites, such as vitamins, cofactors, nucleotides, or amino acids, and are characterized by their ability to recognize their corresponding target molecules with high affinity and specificity without the participation of protein factors [1–5]. This high specificity of riboswitches to their ligands imposes three-dimensional restrictions that result in significant conservation in their primary and secondary structures, even in riboswitches of phylogenetically distant organisms. This conservation is essential to the *in silico* identification of riboswitches within currently sequenced genomes [6–9].

Structurally and functionally, riboswitches are commonly composed of two domains: an aptamer domain, which is part of the riboswitch that selectively recognizes its ligand, and an expression or regulatory domain, which undergoes allosteric modifications due to metabolite binding [7–9]. The level of regulation at which riboswitches act depends on the type of regulatory elements of their expression domain, which mainly include i) premature termination of transcription, mediated by transcriptional attenuators; ii) inhibition of translation initiation by using the formation of mRNA secondary structures in the 5´UTR that sequester the Shine-Dalgarno (SD) sequence or by having the mRNA fragment that results from the premature transcription termination of a riboswitch act *in trans* as a small-antisense RNA that recognizes the SD of the target gene by complementary base pairing; iii) through the reduction in mRNA stability of the regulated gene when the expression platform is folded into an allosteric ribozyme that induces its self-cleavage; and iv) by splicing control, a mechanism that is present only in eukaryotic organisms. In addition to these regulatory mechanisms, riboswitches may block transcription elongation when they are located at the 3´ end of their target genes and are transcribed in an antisense manner.

**Fig 1:**
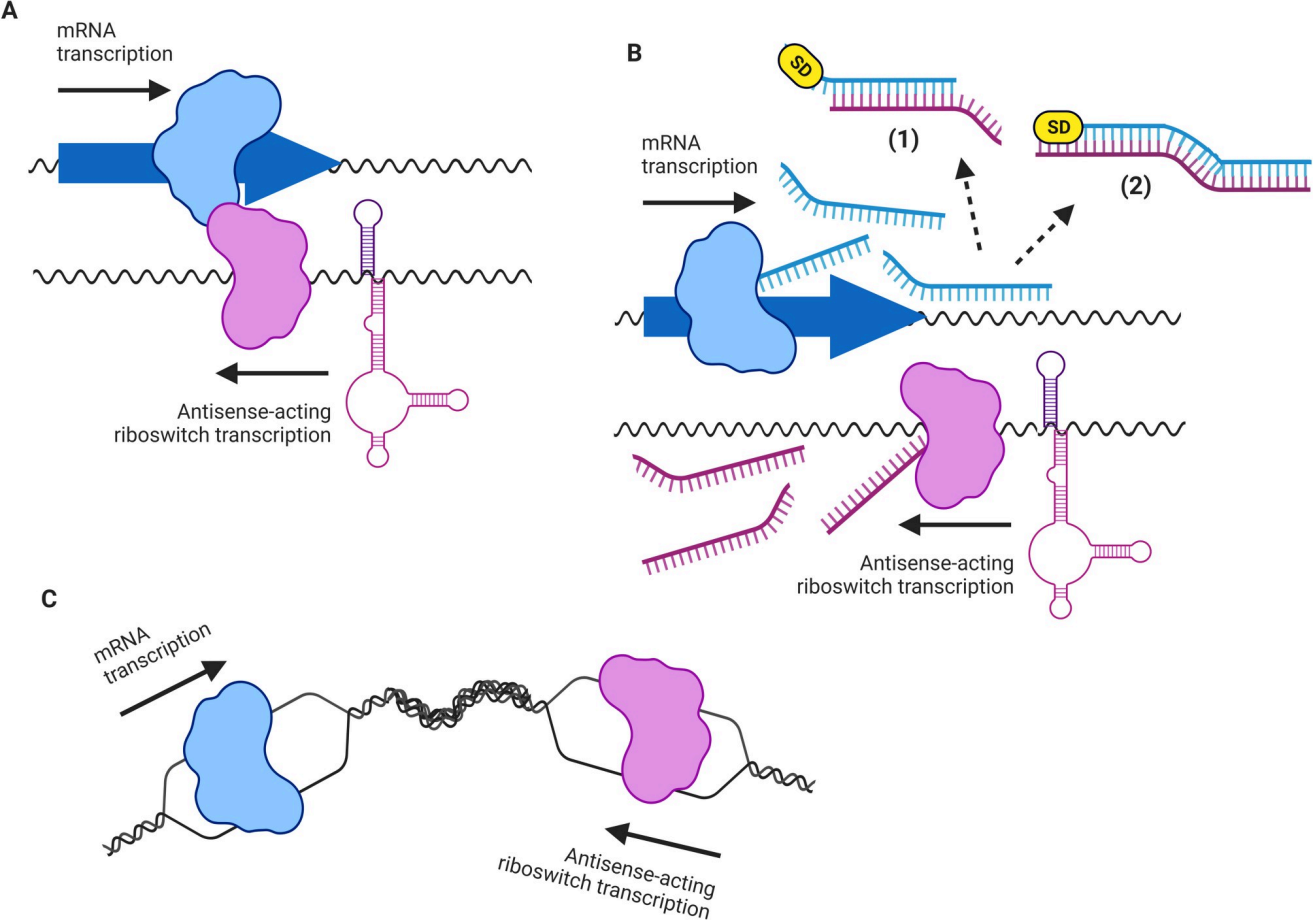
Models of the putative regulatory mechanisms of RNA polymerases transcribing in opposite directions. (A) RNA polymerase collision; (B) asRNA/mRNA duplex formation: (1) partially synthesized double-stranded RNA, (2) totally synthesized double-stranded RNA; (C) DNA supercoil accumulation. Representative elements are colored as follows: mRNA transcription complex, blue; asRNA transcription complex, pink; SD sequence, yellow. Created with BioRender.com.

Antisense RNA-mediated gene regulation has been found to play an essential role in determining the outcome of different biological processes, such as plasmid segregational stability and the control of the plasmid copy number, transposition efficiency, regulation of the lysis/lysogeny phage cycles, and modulation of the toxin-antitoxin system [10]. More recently, the term “excludon” was coined to define a region of DNA whose transcription extends beyond the gene it codes for and continues in an antisense manner within the region of its corresponding adjacent gene, commonly with an opposing function, inhibiting its expression [11]. In principle, three different molecular mechanisms might be involved in the cis*-*antisense RNA regulatory outcome: a) RNA polymerase collision, in which the RNA polymerases from complementary DNA strands collide with each other during convergent transcription; b) duplex antisense RNA formation (asRNA/mRNA), which are subject to degradation by RNase III activity (partially synthesized double-stranded RNA) and inhibition of translation initiation when the SD sequences are sequestered by the antisense mRNA originated from the antisense-acting riboswitch promoter (totally synthesized double-stranded RNA); and c) accumulation of DNA supercoiling, which arises from the positive supercoils in the double DNA strand that are generated as the RNA polymerases co-transcribing in opposite directions move through the same DNA locus [12–14] (Fig 1).

To date, only two examples of antisense-acting riboswitches have been described. The first case, reported in 2008, involves a S-adenosylmethionine (SAM) riboswitch located at the 3’ end that transcribes in the antisense direction of the *ubiG-mccB-mccA* operon in *Clostridium acetobutylicum*. This operon codes for putatively dependent methyltransferase enzymes involved in converting methionine to cysteine. This riboswitch, also known as the S-box, is found in Firmicutes and other Gram-positive bacteria and regulates the expression of genes involved in the biosynthesis, transport, and recycling of methionine [15]. SAM riboswitches commonly regulate their target genes at the level of transcription elongation in response to the intracellular availability of methionine, or its derivatives, through transcriptional attenuators located on their expression platforms. When the intracellular concentration of SAM is high, the regulatory domain is folded into secondary structures that include a transcription terminator, favoring the premature transcription of their regulated genes. Conversely, at low intracellular concentrations of SAM, the formation of a transcriptional antiterminator is induced, and the regulated gene transcription is completed. In the case of the *C*. *acetobutylicum ubiG-mccB-mccA* operon, an opposite regulatory outcome for the SAM antisense-acting riboswitch was described. When the intracellular concentration of SAM in *C*. *acetobutylicum* is low, the formation of a transcriptional antiterminator in the riboswitch expression platform is favored in such a way that the RNA polymerase that transcribes the riboswitch continues toward the interior of the operon in the antisense direction and collides with the RNA polymerase transcribing in the sense direction, resulting in incomplete transcription of the operon and the consequent decrease in the synthesis of its corresponding protein products. In addition, the inhibitory outcome of the transcription coming from the SAM antisense-acting riboswitch might also be generated by the formation of the duplex asRNA/mRNA or the accumulation of DNA supercoiling within the *ubiG-mccB-mccA* locus when the genes are simultaneously transcribed in opposite directions. In contrast, when the intracellular SAM concentration is high, a Rho-independent transcription terminator is formed in the riboswitch’s expression domain, eliminating the interference of antisense-transcribing RNA polymerases. This regulatory use of the SAM riboswitch that transcribes in the opposite direction guarantees that the synthesis of the *ubiG-mccB-mccA* operon occurs only when the intracellular SAM concentration is high [16].

The second reported case of an antisense-acting riboswitch is that of the cobalamin or B_12_ riboswitch of *Listeria monocytogenes* that is located at the 3`end of the *pocR* gene of this organism. PocR is a transcriptional regulator of the genes coding for the enzymes involved in the metabolism of propanediol and thus plays a role in the pathogenic activities of this organism. Since these enzymes require vitamin B_12_ as a cofactor to be active when the intracellular B_12_ concentration is low, the cobalamin antisense-acting riboswitch represses PocR synthesis at the transcription level, due to RNA polymerase collision or the accumulation of DNA supercoiling within the *pocR* gene; at the mRNA stability level, by the formation of an asRNA/mRNA duplex which arises from the transcription of the *pocR* gene in the sense and antisense directions; and also interferes with PocR translation initiation. At a high concentration of vitamin B_12_, a Rho-independent transcriptional terminator is formed in the regulatory domain of the SAM antisense-acting riboswitch, preventing the inhibition of *pocR* expression [17].

Here, we present the results of our comprehensive search for antisense-acting riboswitches in bacterial and archaeal representative genomes. Our findings show that regulation by antisense-acting riboswitches is much more widespread than initially reported, increasing the number from the two cases previously reported in the past decade to 292 biologically significant instances in which the regulatory outcome of antisense-acting riboswitches seems consistent with the metabolite recognized by the riboswitch and the metabolic function of the regulated gene product. Additionally, the genes we identified were classified according to their biological processes into ten categories. A representative example of each of these antisense-acting riboswitch groups is presented, and the metabolic implications of their regulation are discussed in depth.

## Materials and methods

### Identification of riboswitches in genome sequences

To search for riboswitches in the set of 5´ and 3´ intergenic genome sequences, we used the CMsearch software from the Infernal package (v.1.1.3) [18] and the covariance models of the 50 riboswitch classes described in the Rfam database [19]. Since different related covariance models could recognize the same RNA sequence, we used an ad hoc Perl program to select the most suitable model for each identified riboswitch based on their corresponding bit scores reported by the CMsearch program (i.e., c-di-GMP-I, c-di-GMP-II, c-di-GMP-I-GGC, c-di-GMP-II-GAG, c-di-GMP-I-UAU, and c-di-GMP-II-GCG covariance models), which tend to recognize the same set of RNA sequences. The transcription orientations of the riboswitches and their neighbor target genes were defined considering the genomic annotations reported in the KEGG database [20]. We only considered as antisense-acting riboswitches those elements located at no more than 500 nucleotides from their regulatory target genes.

### Selection of representative organism genome sequences

Genome sequences were retrieved from the KEGG database 2022 (https://www.genome.jp/kegg/) with 6,892 bacterial and 380 archaeal organisms. We selected one genome sequence per species to avoid redundant genome sequences, considering those with the highest number of open reading frames. After this selection, the number of genome sequences was 5,086 and 332 for bacterial and archaeal genomes, respectively.

### Statistical analysis of the results

The statistical analysis of the putative antisense-acting riboswitches identified in our study was performed using RStudio software (Version 1.2.5033).

### Analysis of the genome context

The genomic context of the regulated genes and their corresponding antisense-acting riboswitches were analyzed using our GeConT webserver (http://biocomputo.ibt.unam.mx:8080/GeConT/index.jsp) [21].

### Analysis of the metabolic pathways

The metabolic relationships of the enzymes encoded by genes regulated by antisense-acting riboswitches and the metabolites used as riboswitch signals were based on the metabolic pathways of the KEGG pathway database [20].

### Analysis of the gene functions

The function of the regulated genes was analyzed considering the gene descriptions reported in the KEGG database and the description of their corresponding Cluster of Orthologous Genes (COGs). COGs assignations were determined based on a Hidden Markov Models (HMMs) search using the *hmmsearch* program [22] and a set of previously constructed HMM models that represent each of the 4,873 existing COGs [23,24].

### Results and discussion

Within the repertoire of molecular elements that organisms possess to regulate gene expression, riboswitches undoubtedly have a prominent position since they are RNA elements that control the transcription or translation of their target genes in response to the highly specific recognition of their cognate molecules in the absence of protein components, an ability that was thought to be unique to regulatory proteins. To date, 50 classes of riboswitches have been described, according to their covariance models, which recognize small ligands such as metabolites, coenzymes, nucleotides, metal ions, amino acids, and even tRNA molecules, among others. In general, the nature of the genes regulated by riboswitches is related to the biosynthesis or transport of the molecules they recognize. Consequently, the presence of these molecules commonly results in the folding of riboswitch regulatory platforms into secondary structures in the 5’ untranslated region of the mRNA which can promote a premature termination of transcription or inhibit the translation initiation.

To date, two examples of riboswitches have been described whose regulatory activity is contrary to that previously expected. That is, the expression of their corresponding target genes only occurs when the intracellular concentration of their signaling molecules is high. In the first case, a SAM riboswitch located at the 3’ end and transcribing in antisense direction to the *ubiG-mccB-mccA* operon in *C*. *acetobutylicum* guarantees that the synthesis of their protein products occurs only when the intracellular SAM concentration is high. This response is consistent with the fact that the enzymes encoded by the operon above mentioned require this cofactor to be active. Therefore, the expression of these enzymes in metabolic conditions where the cofactor is limited results in futility [16]. The second case involves the regulation of the *L*. *monocytogenes* PocR transcription factor by a Cobalamin (B_12_) riboswitch located at the 3’ end and transcribing in opposite direction to that of the *pocR* gene. In such case, the inhibition of PocR synthesis in the absence of B_12_ can be understood by the fact that the enzymes encoded by the target genes of this transcriptional activator are B_12_-dependent, and the synthesis of cofactor-dependent enzymes is a result of the scarce use in absence of their corresponding cofactors [17]. In the two previous cases, where the riboswitches have inverse regulatory responses to the canonical synthesis/inhibition responses observed in their counterparts in the absence/presence of their target metabolites, two common features can be observed: they are located at the 3’ untranslated region of their mRNAs and their transcription is in opposite direction to that of their target gene. Based on the latter characteristic, we refer to this kind of riboswitch as antisense-acting riboswitches.

### Identification of riboswitches in prokaryotic genomic sequences

We selected a set of genomic sequences from the KEGG 2022 database from 6,892 bacteria and 380 archaea at the species phylogenetic level (see Materials and Methods section). We used the 50 covariance models defined in the Rfam database [25] and the CMsearch program [18] to search for riboswitch elements in the 5’ and 3’ intergenic sequences of the 7,272 prokaryotic organisms and found a total of 99,267 riboswitches (see Materials and Methods for details).

### Identification of putative antisense-acting riboswitches

Considering the direction of transcription of the identified riboswitches in relation to that of their target genes, we initially determined that 98,082 (98.8%) correspond to sense-acting riboswitches and 1,185 (1.2%) to antisense-acting riboswitches. To avoid a statistical bias caused by the overrepresentation in the KEGG database of genomic sequences of microorganisms with several occurrences due to strains of the same species, all subsequent analyzes of our study were performed considering only a representative genomic sequence per species (See Materials and Methods for details).

To find possible trends in which riboswitches might have antisense regulation, we grouped the 50 different classes of riboswitches described in the Rfam database into 27 families according to their shared recognition of their target molecules (S1 Table) (see Materials and Methods section). Subsequently, based on the absolute frequency for each one of the riboswitch families in the sense and antisense orientations, we evaluated the statistical significance of their tendencies to act as antisense-acting riboswitches using a hypergeometric distribution analysis. The results of this analysis are presented in Table 1. What stands out in this table is that the c-di-GMP riboswitch family, commonly involved in cell signaling processes and definition of cell fates such as motility, virulence and biofilm formation, is the one with the highest tendency to regulate its target genes as antisense-acting riboswitches. We also identified a significant tendency for the Cobalamin, ykoK/M-box, and MOCO families, which respond to changing concentrations of cobalamin, magnesium ions and molybdenum, respectively to act as antisense-acting riboswitches. Additionally, the family of riboswitches in which we did not identify antisense regulation are the tetrahydrofolate (THF) and the nickel–cobalt (NiCo) sensing riboswitches.

**Table 1:** Tendency of riboswitch families to act in an antisense orientation.

Riboswitch family	Sense-acting riboswitches absolute frequency	Antisense-acting riboswitches absolute frequency	P-value
c-di-GMP	2,554	152	4.55E-62
Cobalamin	10,769	190	1.05E-10
ykoK / M-box	828	27	1.67E-06
MOCO	834	24	4.34E-05
AAC_AAD	97	3	0.10
glnA	97	2	0.30
nhaA-I	180	2	0.60
sul1	195	2	0.64
Mg_sensor	104	1	0.69
Glycine	3,153	32	0.74
DUF1646	125	1	0.75
raiA	127	1	0.76
ydaO-yuaA	2,799	27	0.81
FMN	3,472	32	0.89
ykkC / Guanidine	2,526	20	0.96
crcB / Fluoride	1,446	9	0.98
pfl / ZMP-ZTP	1,001	5	0.99
TPP	9,766	83	1.00
Purine	2,289	13	1.00
PreQ1	771	2	1.00
glmS	635	1	1.00
Lysine	1,974	8	1.00
SAM	8,039	56	1.00
yybP-ykoY	1,907	5	1.00
THF	290	0	1.00
NiCo	100	0	1.00
T-box	15,851	107	1.00

### Classification of the types of genes regulated by antisense-acting riboswitches

To elucidate possible trends in which antisense-acting riboswitches regulate the expression of genes, and to understand the nature of the biochemical implications of such regulation, we classified antisense-regulated genes into ten groups: a) genes coding enzymes involved in the interconversion of a compound related to the metabolite sensed by the riboswitch; b) genes coding enzymes that require a cofactor sensed by the riboswitch to become active or to participate in a metabolic pathway wherein another enzyme requires the cofactor; c) genes coding transporter proteins for compounds related to the metabolite sensed by the riboswitch; d) genes coding cell signaling proteins; e) genes coding transcriptional regulators; f) genes coding transposases; g) pseudogenes; h) uncharacterized ORFs with clear orthologs in multiple genomes; i) hypothetical small genes without orthologs; and j) other types of genes. Table 2 shows the absolute and relative frequency of genes potentially regulated by the antisense-acting riboswitches which belong to the different groups above-mentioned.

**Table 2:** Frequency of genes potentially regulated by antisense-acting riboswitches.

Gene classification	Absolute frequency of genes regulated by antisense-acting riboswitches	Relative frequency (%)
Interconversion	116	14.41
Cofactor	25	3.11
Transporters	69	8.57
Signaling	12	1.49
TFs	70	8.70
Transposase	54	6.71
Pseudogene	50	6.21
Uncharacterized	57	7.08
Hypothetical	274	34.04
Other	78	9.69

### Biochemical outcomes of antisense-acting riboswitch regulation

To exemplify each of the different groups of genes identified as being regulated by antisense-acting riboswitches, we chose a representative example of each group. Then, we analyzed in detail the possible biochemical implications of their regulation. S3 Table shows the genes identified and their corresponding groups.

#### a) Genes encoding enzymes involved in the interconversion of compounds related to the metabolite sensed by the riboswitch

**Fig 2:**
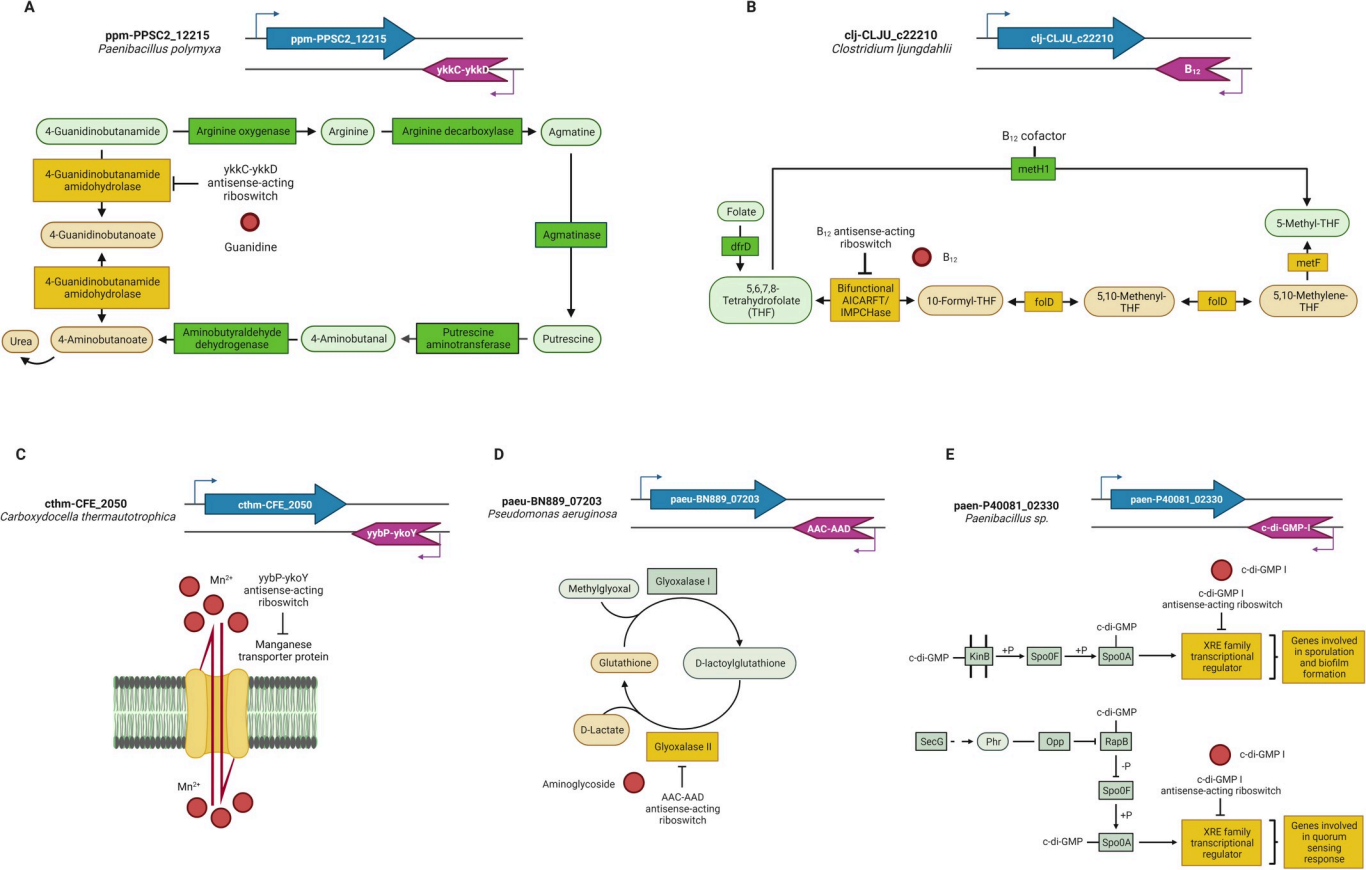
Representative pathways of genes regulated by antisense-acting riboswitches. (A) Gene encoding an enzyme involved in the interconversion of a compound related to the metabolite sensed by the riboswitch; (B) Gene encoding an enzyme that requires a cofactor sensed by the riboswitch to become active or participates in a metabolic pathway wherein another enzyme requires the cofactor; (C) Gene encoding a transporter protein for a compound related to the metabolite sensed by the riboswitch; (E) Gene that codes for a transcriptional regulator. Metabolic route active in the presence (yellow) or absence (green) of the metabolite sensed (red circle) by the antisense-acting riboswitch. Representative elements are colored as follows: Target genes, blue; antisense-acting riboswitch, violet. Enzymes are indicated by rectangles; compounds are indicated by ovals. Created with BioRender.com.

We identified a ykkC-ykkD or Guanidine-I [26] antisense-acting riboswitch in the *Paenibacillus polymyxa* SC2 genome that regulates the ppm-PPSC2_12215 gene that encodes an amidase protein, AmyE (Fig 2A). This enzyme, better known as 4-guanidinobutanamide amidohydrolase, is involved in the arginine metabolism pathway catalyzing the conversion reaction from 4-guanidinobutanamide into 4-guanidinobutanoate, resulting in the conversion of an amide into a carboxylic acid functional group.

Arginine is the core compound of this pathway, and it acts as a precursor in the biosynthesis of several polyamines, such as putrescine and spermidine, which are essential in several physiological processes. It can also be a poor nitrogen source [27]. Furthermore, arginine is the only amino acid with a guanidine group in its composition, despite the fact that it comprises compounds that are prominent within bacterial cells, including creatinine and secondary metabolites such as streptomycin [28–30].

The antisense-acting riboswitch identified at the 3´ end of the ppm-PPSC2_12215 gene would enable the synthesis of the 4-guanidinobutanamide amidohydrolase enzyme when guanidine is present; in this case, its availability allows the progression along the pathway while being a part of 4-guanidinobutanamide, a compound that is catabolized into butanoate and urea in a subsequent step. On the other hand, when the riboswitch senses no guanidine available, the pathway is interrupted to maintain the transient guanidine levels while being a part of the 4-guanidinobutanamide compound.

As expected, the genes in this group that are regulated by active antisense-acting riboswitches are those that encode enzymes with transferase activities such as aminotransferases, phosphatases, acyltransferases, synthetases, and dehydrogenases. The regulation of the antisense-acting riboswitches of this group inhibits the synthesis of their corresponding target genes when they detect low concentrations of the substrates involved. The most important families of active antisense riboswitches in this group mainly include the cobalamin, TPP, T-box, SAM, FMN, and MOCO riboswitches (see S3 Table).

#### b) Genes coding enzymes that require a cofactor sensed by the riboswitch to become active or to participate in a metabolic pathway wherein another enzyme requires the cofactor

A cobalamin antisense-acting riboswitch regulating the clj-CLJU_c22210 gene that encodes the bifunctional phosphoribosylaminoimidazolecarboxamide formyltransferase/IMP cyclohydrolase enzyme, better known as the AICARFT/IMPCHase enzyme, was identified in the *Clostridium ljungdahlii* DSM 13528 genome through our bioinformatic analysis. *C*. *ljungdahlii* is an anaerobic acetogenic bacterium that ferments sugar or CO_2_, that synthesizes gas (CO/H_2_), and that can utilize CO as a substrate. Moreover, this organism possesses a significant number of proteins that require cobalamin as a cofactor and is also able to synthesize cobalamin via an anaerobic pathway [31,32].

The AICARFT/IMPCHase enzyme is involved in the one-carbon metabolism pathway, which comprises the folate and methionine cycles and allows the generation of methyl groups that are used in different methylation reactions and in the biosynthesis of important anabolic precursors, one of which is 5-methyl-tetrahydrofolate (5-methylTHF), whose synthesis can be carried out through two different routes. The starting point of both is tetrahydrofolate (THF) synthesis from folate by the activity of the dihydrofolate reductase enzyme DfrD. In one route, THF is converted into 10-formylTHF by the bifunctional AICARFT/IMPCHase enzyme, which encodes a gene regulated by the cobalamin antisense-acting riboswitch identified. Then, 10-formylTHF acts as the substrate for the synthesis of 5,10-methenylTHF and 5,10-methyleneTHF in two consecutive reactions catalyzed by the bifunctional methylenetetrahydrofolate dehydrogenase (NADP+)/methenyltetrahydrofolate cyclohydrolase enzyme, FolD. This last compound is the precursor for the synthesis of 5-methylTHF catalyzed by methyleneTHF reductase enzyme, MetF, with the participation of NADPH and flavoproteins (Fig 2B). Additionally, through another route, 5-methylTHF is directly synthesized from THF by the methionine synthase enzyme MetH1, whose activity is cobalamin-dependent (Fig 2B).

The cobalamin antisense-acting riboswitch identified regulates the clj-CLJU_c22210 gene, which codes for the bifunctional AICARFT/IMPCHase, which allows the selection of the route that will be used for the synthesis of 5-methylTHF, according to the bacterial cobalamin availability. We propose that when the intracellular concentration of cobalamin is high, 5-methylTHF can be directly synthesized from THF by the cobalamin-dependent enzyme MetH1 (Fig 2B), while when there are changes in the intracellular concentration of cobalamin, the riboswitch-dependent regulation mechanism will favor the activity of the bifunctional AICARFT/IMPCHase enzyme. By these means, the regulation of the clj-CLJU_c22210 gene by the cobalamin antisense-acting riboswitch guarantees 5-methylTHF biosynthesis and reassures the correct cobalamin utilization due to its metabolically expensive biosynthesis and uptake. In addition, the metabolic intermediates of this route have an essential role in the biosynthesis of vital molecules such as purines, DNA, CoA, and serine (Fig 2B) [32,33].

Due to the nature of the genes regulated in this group, which are characterized as encoding enzymes that require a cofactor to be active, the family of cobalamin antisense-acting riboswitches (Cobalamin, AdoCbl, and AdoCbl-variant) is the most important member of this group, since this family represents 38% of the total number of riboswitches in this category. In general, the families of enzymes with isomerase and methyltransferase activities are the ones that most frequently require cobalamin, or some of its derivatives, as cofactors. Another important family of antisense-acting riboswitches in this group is the TPP family. The TPP cofactor consists of a pyrimidine ring, a thiazole ring, and a pyrophosphate functional group, which are connected to each other with the thiazole ring being the portion commonly involved in enzymatic reactions, as it is the most reactive part of the molecule. We also found examples of molybdenum cofactor (MOCO) antisense-acting riboswitches that regulate genes involved in the metabolism of molybdenum and tungsten cofactors (S3 Table) [34].

#### c) Genes encoding transporter proteins for compounds related to the metabolite sensed by the riboswitch

An antisense-acting yybP-ykoY [26] riboswitch, whose binding metabolite is manganese (Mn^2+^), regulates the cthm-CFE_2050 gene that encodes an Mn^2+^ transporter protein in the *Carboxydocella thermautotrophica* 019 genome (Fig 2C). Mn^2+^ is an essential ion in bacterial metabolism that participates in several metabolic processes, acting mainly as a cofactor in various enzymes and as a protective agent against reactive oxygen species (ROS) through the transient substitution of Mn^2+^ for iron (Fe) molecules in the active sites of some enzymes to prevent the oxidative damage of proteins [35]. On the other hand, excess Mn^2+^ can be toxic since its accumulation in bacteria is directly related to a deficiency of other ions, which can then provoke the incorrect metalation of transcription factors and critical enzymes, affecting cellular development, virulence, and ROS sensitivity [26,36–39].

The gene identified as subject to regulation by this antisense-acting riboswitch is extensively distributed throughout the phylum Firmicutes and has an essential role in *C*. *thermautotrophica* 019 metabolism. This bacterium has been defined as carboxydotrophic, but its metabolic capacities remain mostly unknown. Nevertheless, as the ability to transport Mn^2+^ has been observed in other species, it is known that these species can proliferate in hostile environments, including over mineral substrates compounded by Mn^2+^, such as diatomite and glauconite, in the absence of a carbon source [40,41]. The antisense-acting riboswitch identified at the 3´ end of the cthm-CFE_2050 gene would prevent the excessive internal accumulation of the Mn^2+^ that would otherwise have toxic effects.

Many genes of this third group are members of the ABC-type transport systems or code for different transporter proteins, such as the Co/Zn/Cd cation transporters, the Na^+^/H^+^-dicarboxylate symporters, or the Ca^2+^/Na^+^ antiporters. The most common antisense-acting riboswitches of this group are the cobalamin, c-di-GMP-I, T-box, SAM, ykoK, TPP, and MOCO riboswitches (see S3 Table).

#### d) Genes coding cell signaling proteins

An AAC-AAD antisense-acting riboswitch, which senses and responds to aminoglycoside concentrations [42], was found to regulate the paeu-BN889_07203 gene that codes hydroxyacylglutathione hydrolase enzyme synthesis in *Pseudomonas aeruginosa* PA38182 (Fig 2D). This enzyme, better known as glyoxalase II (GlxII), participates in the methylglyoxal (MG) metabolism pathway through the glyoxalase (Glx) system, which is an enzymatic complex for critical detoxification. It comprises two metalloenzymes in charge of breaking down cytotoxic MG, metabolizing it into its corresponding α-hydroxy acids.

The first enzyme of this system is glyoxalase I (GlxI) or S-D-lactoyl-glutathione methylglyoxal lyase, which is an isomerizing divalent reductase that can be Zn^2+^-dependent or non-Zn^2+^-dependent (activated by Ni^2+^/Co^2+^). GlxI accepts MG as a glutathione hemiacetal (GSH) and catalyzes proton transfer, transforming GSH into its corresponding thioester, lactoylglutathione. The second enzyme, GlxII, regenerates the thiol cosubstrate GSH and the α-hydroxy acid lactate in the case of MG detoxification. MG is highly toxic to cell reactive electrophilic species (RES) producer molecules, and they produce cellular stress through reactions with nucleophilic macromolecules. MG also acts as a transcriptional activator and participates in the kinase activation cascade, having a primary role in regulation processes or stress adjustment conditions, making it an essential signaling molecule [43,44].

During the *P*. *aeruginosa* pathogenic process, the bacteria are first attacked by the innate immunologic system, primarily via phagocytes. Within the phagocytes, ROS are generated in an NADPH oxidase-dependent manner as bactericidal substances. Thus, bacterial cells are exposed to oxidative stress, inducing damage due to interactions with cellular elements such as lipids, DNA, and proteins. This process leads to lipid peroxidation, DNA mutation, DNA‒protein cross-linking, protein oxidation, and breakage. *P*. *aeruginosa* has evolved mechanisms to protect itself and survive under these hostile conditions. One of them is the production of GSH, the most abundant antioxidant molecule within the cells, which plays a relevant role in ROS removal, acting as an electron donor. GSH is also involved in cellular homeostasis preservation, sulfur transport regulation, metabolite combinations, xenobiotic detoxification, antibiotic resistance, enzymatic regulation, and stress response gene expression [45,46].

Compounds that produce cellular oxidative stress and their effects on different bacterial developmental stages have been widely studied. These compounds increase the aminoglycoside sensitivity of *P*. *aeruginosa* by disrupting membrane permeability, mainly during biofilm production, which is stimulated by sublethal antibiotic concentrations, such as tobramycin and gentamycin aminoglycosides. In addition, oxidative stress acts as a signal for the expression of efflux systems, which promotes bacterial antibiotic resistance [47–49].

The antisense-acting riboswitch identified at the 3´ end of the para-BN889_07203 gene would enable the gene expression of the GlxII enzyme when the aminoglycoside is sensed in the environment. On the other hand, when no aminoglycoside is present in its medium, the expression of the enzyme is repressed, as the bacteria are not subject to oxidative stress from the antibiotic presence.

This fifth group of genes codes for proteins that are mainly involved in different stress responses and developmental processes, such as quorum-sensing autoinducer synthesis, and for different kinds of signal transduction histidine kinases. The principal antisense-acting riboswitches of this group are those that sense the cyclic dinucleotide second messengers c-di-GMP-I and cyclic di-AMP (YdaO/YuaA) riboswitches (S3 Table) [50].

#### e) Genes coding transcriptional regulators

We identified an antisense-acting c-di-GMP-I riboswitch [51] regulating the paen-P40081_02330 gene coding a transcriptional regulator of the XRE family in the *Paenibacillus* sp. FSL P4 genome (Fig 2E). This transcriptional regulator, whose nearest homolog is SinR, a transcriptional repressor known as the master regulator for biofilm formation in *Bacillus subtilis*, controls the expression of genes involved in exopolysaccharide, matrix protein production, and motility.

The synthesis of signaling molecules is one strategy bacteria employ to sense alterations in their environment in order to rapidly adjust according to those changes. One of these molecules is the bis-(3′-5′)-cyclic dimeric GMP (c-di-GMP), which is able to induce the transition from a unicellular motile state to a multicellular sessile state through phosphorelay systems that control biofilm and spore formation (Fig 2E) [52–55].

For many bacteria, the quorum-sensing intercellular communication mechanism is essential for gene expression pattern coordination at the population level. Furthermore, for motility, biofilm matrix production, and spore formation processes, quorum sensing is critical, as it is one of the main strategies to sense and respond to environmental changes (Fig 2E) [56–59].

The c-di-GMP-I antisense-acting riboswitch regulation of a transcription factor coded by the paen-P40081_02330 gene that we propose is based on the detection of this molecule, triggering the expression of the genes involved in biofilm and spore formation, as well as quorum-sensing processes, and having a general effect on their expression when it is not sensed, thanks to its close relationship with the master regulator of these processes, Spo0A (Fig 2E).

As mentioned above, in the literature, there are only two examples of the regulation of gene expression mediated by riboswitches that act in an opposite direction, one of which is the cobalamin antisense-acting riboswitch identified at the 3`end of the gene encoding the PocR transcriptional regulator of the enzymes involved in the metabolism of propanediol in *L*. *monocytogenes*, the enzymes of which require cobalamin as a cofactor to be active. In our study, we identified cobalamin antisense-acting riboswitches in the orthologous *pocR* genes of other Listerias and in the Enterococcaceae *Vagococcus carniphilus*. The regulation of genes encoding transcription factors (TFs) makes it possible to extend the regulatory outcome of the riboswitches that act in an opposite direction to multiple functionally related genes in different operons, which have in common the operator site of the corresponding TFs. In our study, the c-di-GMP-I, cobalamin, and TPP antisense-acting riboswitches were the most frequent riboswitches of this group.

#### f) Genes coding transposases

**Fig 3:**
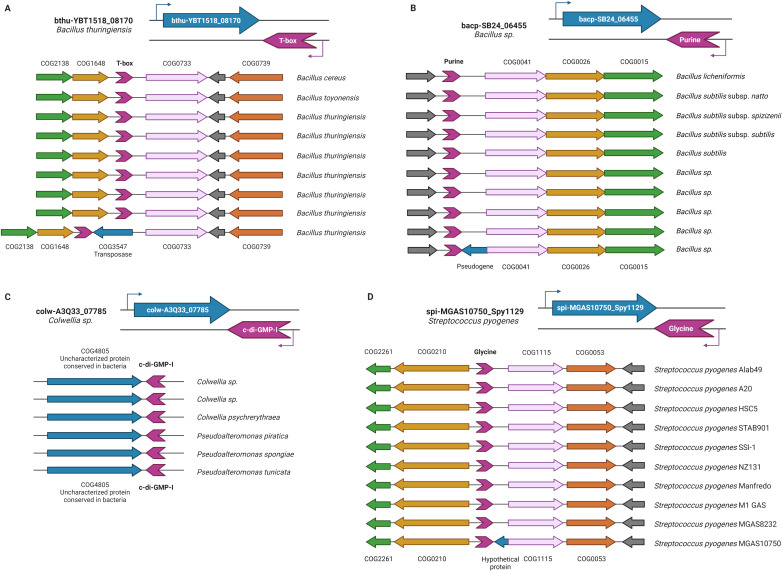
Representative syntenic context of genes regulated by antisense-acting riboswitches. The upper panel on each section shows in detail an example of the different types of genes regulated by the antisense-acting riboswitches, while the lower panel shows the context in closely related organisms compared to the example. (A) Gene coding for a transposase: COG2138-Uncharacterized conserved protein, COG1648-Siroheme synthase, COG0733-Na+-dependent transporters of the SNF family, COG0739-Membrane proteins related to metalloendopeptidases; (B) Pseudogene: COG0041-Phosphoribosylcarboxyaminoimidazole (NCAIR) mutase, COG0026-Phosphoribosylaminoimidazole carboxylase (NCAIR synthetase), COG0015-Adenylosuccinate lyase; (C) Gene coding for an uncharacterized ORF with clear orthologs in multiple genomes; (D) Hypothetical small gene without orthologs: COG2261-Predicted membrane protein, COG0210-Superfamily I DNA and RNA helicases, CO1115-Na+/alanine symporter, COG0053-Predicted Co/Zn/Cd cation transporters. Representative elements are colored as follows: Riboswitch spuriously regulated genes, blue; antisense-acting riboswitch, violet; *bona fide* regulated gene, pink. Genes are colored according to the COG they belong to. Created with BioRender.com.

Unlike the aforementioned examples, where regulation of gene expression mediated by antisense-acting riboswitches represents a selective advantage, we propose that in the case of transposon-encoding genes, the existence of antisense-acting riboswitches corresponds to the random insertion of the transposable element in chromosome regions where the riboswitches were previously regulating other genes in a canonical manner. Consistent with this hypothesis, Fig 3A shows the genomic context of the bthu-YBT1518_08170 gene in phylogenetically closely related organisms. In the genomes of all these organisms, except for that of the *Bacillus thuringiensis* YBT-1518, the T-box riboswitch regulates the downstream gene coding for a tryptophan transporter in a canonical manner.

In accordance, we evaluated the frequency with which the gene located upstream of transposase has an orthologous gene regulated by the same antisense-acting riboswitch. S4 Table shows that our hypothesis is consistent with their genomic context by over 50% of the cases of genes that code for transposases.

#### g) Pseudogenes

Another trend that we observed in our analysis was the presence of antisense-acting riboswitches next to ORFs annotated as pseudogenes, such as the bacp-SB24_06455 gene of *Bacillus sp*. Pc3 (Fig 3B). In a similar manner, as mentioned above, we performed a genome context analysis of the antisense-regulated gene and compared it to its nearest related organism genes. Based on the observed synteny conservation in all the genes, except in the bacp-SB24_06455 gene, we propose that the existence of an antisense-acting riboswitch does not correspond to a selective advantage for the organisms, but rather to a fortuitous event that might be related to the origin of the pseudogene (S5 Table).

#### h) Uncharacterized ORFs with clear orthologs in multiple genomes

The next category of genes regulated by antisense-acting riboswitches identified in our study corresponds to ORFs annotated as "uncharacterized” but with *bona fide* orthologs in other genomes. We included in this group genes larger than 300 nucleotides only (see S3 Table). An example of a gene that belongs to this category is colw-A3Q33_07785 of *Colwellia* sp. PAMC 21821, whose function is still unknown (Fig 3C).

#### i) Hypothetical small genes without orthologs

Genes identified as potentially regulated by an antisense-acting riboswitch included in this category are characterized by having small sizes of less than three hundred bases and lacking apparent orthologs in the set of sequenced genomes, accounting for 34% of the elements. These characteristics allow us to consider that these "hypothetical genes" correspond to probable annotation errors of open reading frames that, in most cases, are incorrectly annotated as genes. As in our previous group, the comparative analysis of syntenic regions in phylogenetically closely related organisms confirms our hypothesis that most of those riboswitches (72% of our studied cases) regulate the neighboring upstream gene of the hypothetical gene, according to the consistency of its genomic context. This is the case for the hypothetical small gene spi-MGAS10750_Spy1129 in the *Streptococcus pyogenes* MGAS10750 genome with a Glycine riboswitch at its 3´ end and a D-alanine glycine permease gene, whose orthologous counterparts are regulated by Glycine riboswitches in phylogenetically related organisms (Fig 3D). In S6 Table, we list all these hypothetical genes as well as the most likely regulated gene neighbor.

#### j) Other types of genes

Finally, the last group in our analysis is composed of antisense-acting riboswitches, identified next to genes that could not be classified into any of the previously described groups. The genes in this category represent approximately 9% of the antisense riboswitches identified in our study, and the biochemical implications of their regulation are not obvious at first glance.

### Genes regulated by tandem antisense-acting riboswitches

Tandem riboswitches have been found to regulate gene expression in several organisms [60]. They might include either two or more copies of the same kind of riboswitch to extend the range of regulation by the sensing signal, or copies of different riboswitch classes next to those genes that require a more complex regulation which obeys to more than one intracellular signal [60]. In our study, we searched for tandemly arranged antisense-acting riboswitches and found seven genes potentially regulated by elements with this characteristic (S7 Table); four of them correspond to transposase genes, two of them to ABC-transporter operons, and the last one to a small hypothetical gene of 126 nucleotides in length. As described for our classification of small hypothetical genes, these commonly correspond to annotation errors. In accordance with this hypothesis, the upstream genes to a small hypothetical gene regulated by an antisense-acting riboswitch tend to be regulated by the same riboswitch family in orthologous genes which do not have such small hypothetical gene. In the *Ralstonia insidiosa* FC1138 sequence genome, we identified a small gene, rin-ACS15_3586, annotated as hypothetical, with a tandemly arranged couple of glycine antisense-acting riboswitches. Upstream of this gene, we found the *gcvT-gcvH-gcvP* operon, coding for the glycine cleavage system T and H proteins, and the HNH endonuclease family protein, respectively, which are genes commonly regulated by glycine riboswitches in other organisms.

About a decade ago, when only two examples of antisense-acting riboswitches had been described, the number of publicly available and fully sequenced genomic sequences from prokaryotic organisms was barely one hundred. Today, we have tens of thousands of newly sequenced genomes. The question, then, is why no other examples of this kind of noncanonical riboswitch have been reported. Are there no more examples of antisense-acting riboswitches in orthologous genes or other kinds of regulatory systems involving enzymes that require cofactors analogous to those mentioned above? What new metabolic conditions might require regulation based on antisense-acting riboswitches that could be identified in a global study using currently available genomic sequences? These are the main questions that motivated our study. As a result of our analysis, we identified 292 antisense-acting riboswitches whose regulatory outcomes seem biologically significant. Analysis of these potentially antisense action riboswitches allowed us to classify them into ten different groups according to the nature of the genes they regulate or a common characteristic shared among them. Our results are consistent with our biochemical understanding of the requirements of organisms under different metabolic conditions and how these requirements are satisfied by the regulatory responses of genes subject to antisense-acting riboswitch responses. Additionally, it is important to note that our method was able to identify the two antisense-acting riboswitches previously described. Our findings concerning antisense-acting riboswitches provide evidence of the evolutionary plasticity that organisms have developed to obtain opposite regulatory responses using the same regulatory elements, but only modifying their location (5’ or 3’ UTR) and transcription orientation (sense or antisense) according to their target genes.

## Supporting information

S1 Table Riboswitch families. (XLSX)

S2 Table Antisense-acting riboswitches. (XLSX)

S3 Table Antisense regulated genes. (XLSX)

S4 Table Transposases. (XLSX)

S5 Table Pseudogenes. (XLSX)

S6 Table Hypothetical proteins. (XLSX)

S7 Table Genes regulated by tandem antisense-acting riboswitches. (XLSX)

S1 File (ZIP)
